# Amino Acid Transport and Metabolism in Myeloid Function

**DOI:** 10.3389/fimmu.2021.695238

**Published:** 2021-08-12

**Authors:** Marie Jo Halaby, Tracy L. McGaha

**Affiliations:** ^1^Tumor Immunotherapy Program, Princess Margaret Cancer Centre, University Health Network, Toronto, ON, Canada; ^2^Department of Immunology, The University of Toronto, Toronto, ON, Canada

**Keywords:** macrophage, MDSC, amino acid, IDO - indoleamine 2 3-dioxygenase, arginase (ARG), immune suppression, inflammation

## Abstract

Regulation of amino acid availability and metabolism in immune cells is essential for immune system homeostasis and responses to exogenous and endogenous challenges including microbial infection, tumorigenesis and autoimmunity. In myeloid cells the consumption of amino acids such as arginine and tryptophan and availability of their metabolites are key drivers of cellular identity impacting development, functional polarization to an inflammatory or regulatory phenotype, and interaction with other immune cells. In this review, we discuss recent developments and emerging concepts in our understanding of the impact amino acid availability and consumption has on cellular phenotype focusing on two key myeloid cell populations, macrophages and myeloid derived suppressor cells (MDSCs). We also highlight the potential of myeloid-specific of amino acid transporters and catabolic enzymes as immunotherapy targets in a variety of conditions such as cancer and autoimmune disease discussing the opportunities and limitations in targeting these pathways for clinical therapy.

## Introduction

Myeloid cells are a broad subset of immune cells essential for innate immune responses against infection and injury. Mobilized to infection sites, myeloid populations play key roles in elimination of pathogens. In addition to this direct role in responses to infection, myeloid cells are essential for tissue homeostasis, remodelling, wound repair and scar tissue formation. This crucial function in tissue maintenance has a darker side as myeloid cells also are key drivers of pathophysiology in diseases of chronic inflammation like cancer and autoimmunity. Although interest in myeloid cells in this context has significantly increased as of late, we understand relatively little regarding the signals that influence myeloid fate or function in the local tissue milieu. A concept that has received significant attention is the impact of metabolism and microenvironmental influence on myeloid function. In particular, metabolic/enzymatic consumption of amino acids is a core aspect of myeloid biology as acquired polarization states in myeloid cells are closely associated with increased expression of enzymes that target key amino acids like arginine and tryptophan. Importantly, the data now show that that amino acid metabolization and the local microenvironmental availability of amino acids is an integral component in myeloid development, migration, polarization, and turnover likely impacting a wide range of disease states. In this review, we will focus on two key populations of myeloid cells involved in pathology of diseases of chronic inflammation, macrophages and MDSCs, examining how amino acid availability and metabolism affects their function under homeostatic and pathologic conditions.

Macrophages are the most abundant myeloid cell found in most tissues. Named for the ability to phagocytize large amounts of particulate matter, macrophages are essential for wound healing responses (1) and are enriched in many tumor types where they play varied roles related to stromal support for the neoplastic cells and immune suppression in the tumor microenvironment (2). Likewise, we and others have shown that macrophages are key drivers of immune regulation limiting autoimmune disease pathology *via* their ability to influence local and systemic inflammatory responses (3, 4). Macrophages are functionally plastic and can exhibit alternate, often opposing characteristics driven by complex combinations of signals dependent on tissue of residence, microenvironmental conditions encountered in the tissue, and developmental origin (albeit developmental influence is still poorly understood). Macrophages do not represent a single cellular lineage; however, they are often described by a binary functional phenotype based in inflammatory or immune suppressive potential (i.e. M1-like versus M2-like respectively). The M1-like phenotype is characterized by prominent production of pro-inflammatory mediators such as the cytokines IL-1β, IL-6, IL-12 and TNFα, and small molecules such as nitric oxide (NO) that amplify inflammation. In opposition to this, macrophage exposure to immune regulatory cytokines (e.g. IL-4, IL-10, IL-13, TGFβ1) or immunoglobulin/antigen immune complexes drives the anti-inflammatory M2-like phenotype. M2-like polarization is associated with induction of IL-10 and TGF-β expression, the chemokines CCL17 and CCL22 and CCL17, and the enzyme arginase 1. In vivo however, it is unlikely that a pure M1 or M2 macrophages polarization state exist, and tissue macrophages display a mixture of traits reflective of the complex milieu they encounter in the microenvironment.

Myeloid-derived suppressor cells (MDSC) were first identified in cancer patients and tumor-bearing mice, where they were found to be significantly increased (up to ten-fold) in the circulation and peripheral lymphoid organs compared to healthy individuals. MDSCs are distinctive in the fact that they appear to be locked in an immature proliferative state distinguished from their mature counterparts by expression of immature stem-like markers and potent immune suppressive function (5). MDSCs are not specific cell lineage, but rather are a mixed population of cells broadly classified by morphological features and patterns of surface receptor expression resembling polymorphonuclear cells (PMN-MDSCs) or monocytes (M-MDSCs). In mice, PMN-MDSCs are CD11b^+^ Ly6C^lo^ Ly6G^+^, while M-MDSCs are CD11b^+^ Ly6C^hi^ Ly6G^-^; while in humans, PMN-MDSCs are lin^-^HLA-DR^-^CD33^+^CD11b^+^CD14^-^CD15^+^, whereas M-MDSCs are lin^-^HLA-DR^-^CD33^+^CD11b^+^CD14^+^CD15^-^ (6). Of particular relevance to this review, MDSC activation drives high expression of amino acid metabolization enzymes which are key drivers of MDSC immunosuppressive properties (5).

Immune cells utilize distinct metabolic pathways depending on need and functionality. In this vein macrophages exhibit very different metabolic profiles depending on the polarization state and tissue site of residence (7). In a broad description, inflammatory macrophages rely mainly on glycolysis for energy production, whereas tissue resident macrophages rely more heavily on oxidative phosphorylation, glutamine metabolism and fatty acid oxidation to meet their energy needs (8). This is in contrast to MDSCs which display more of a mixed metabolic phenotype with both a more glycolytic profile and a reliance on glutamine for function (9). Particularly, in the tumor microenvironment, MDSCs show increased glycolysis compared to neutrophils in a normal setting that has been linked to the accumulation of intratumoral MDSCs (10). However, the shift to glycolysis in intratumoral MDSCs may also alter mechanisms of immunosuppression as it limits reactive oxygen species (ROS) accumulation by decreasing the reliance of MDSCs on OXPHOS and by the production of phosphoenolpyruvate (10).

## Arginine Catabolism and Innate Immunity

Arginine metabolism is a key regulator of myeloid function, and the mode of arginine consumption is often indicative of the functional state. Arginine is primarily catabolized by two enzymes families, arginase [i.e.arginase-1 and 2 (Arg1, Arg2)] and nitric oxide synthase [i.e. nitric oxide synthase 1 and 2 (Nos1 and Nos2)]. Nos2 (also known as iNos) is the main Nos isoform expressed in myeloid cells, while Arg1 is the dominant arginase isozyme found in immune cells (11). In response to pro-inflammatory NF-κB signaling, Nos2 expression is strongly induced converting arginine to nitric oxide (NO) and citrulline, conferring cytotoxic ability to inflammatory macrophages (12). This activity requires a steady flow of extracellular arginine into the cell. If arginine availability is low, citrulline can be converted back into arginine by arginosuccinate synthase 1 and arginase lyase 1, which can then be used to synthesize more nitric oxide (11). In contrast to Nos2, Arg1 is the predominant catabolic enzyme of arginine in alternatively activated macrophages. It is induced by a number of cytokines such as IL-10, IL-4 and IL-13 (13) as well as phagocytosis of apoptotic cells (14), converting arginine into ornithine and urea. Ornithine is a key building block in wound healing by conversion into proline *via* ornithine aminotransferase, which promotes type-I collagen synthesis and extracellular matrix deposition (11).

Arginine is a conditionally essential amino acid which can be synthesized by the body; however, under situations of high metabolic activity or other states of heightened consumption the need can quickly outstrip the supply. Accordingly, in situations of high arginine catabolism (e.g. when myeloid cells induce expression of Arg1 or Nos2), local arginine availability becomes a limiting factor for immune function. An example of this is T cells, which require arginine for activation, proliferation, and survival (15, 16). Uptake of Arginine by T cells is required for acquisition of a central memory phenotype, promoting potent immunity with increased longevity (17). In the tumor microenvironment, a primary mechanism of MDSC-mediated suppression of T cell function is *via* depletion of arginine from the microenvironment by virtue of Arg1/Nos2 activity (5). While both PMN-MDSCs and M-MDSCs are potently capable of suppressing T cell proliferation *via* pathways that require arginine consumption, they do so by distinct mechanisms. PMN-MDSCs exert immune suppressive activity through Arg1 activity, whereas M-MDSCs suppress T cell function *via* Nos2 dependent mechanisms (5, 18). Recently, an additional mechanism of arginine mediated MDSC immune suppressive activity was described. MDSCs, but not other immune cells, produce a dicarbonyl compound, methylglyoxal. Methylglyoxal is released in close proximity to neighboring T cells, where it prevents arginine accumulation, thereby inhibiting activation and production of inflammatory cytokines such as IFNγ (19). In addition to cancer, an increase in MDSCs has been observed in autoimmune disorders (5, 20). Although the function is not clear, it has been reported that PMN-MDSCs and Arg1 activity are significantly upregulated in SLE patients compared to healthy controls. Interestingly, these patients displayed increased TH17 cells and cytokines, and this increase was found to be dependent on Arg1 activity indicating MDSCs and arginine metabolism are vital for the progression of SLE pathophysiology (20).

**Figure 1:**
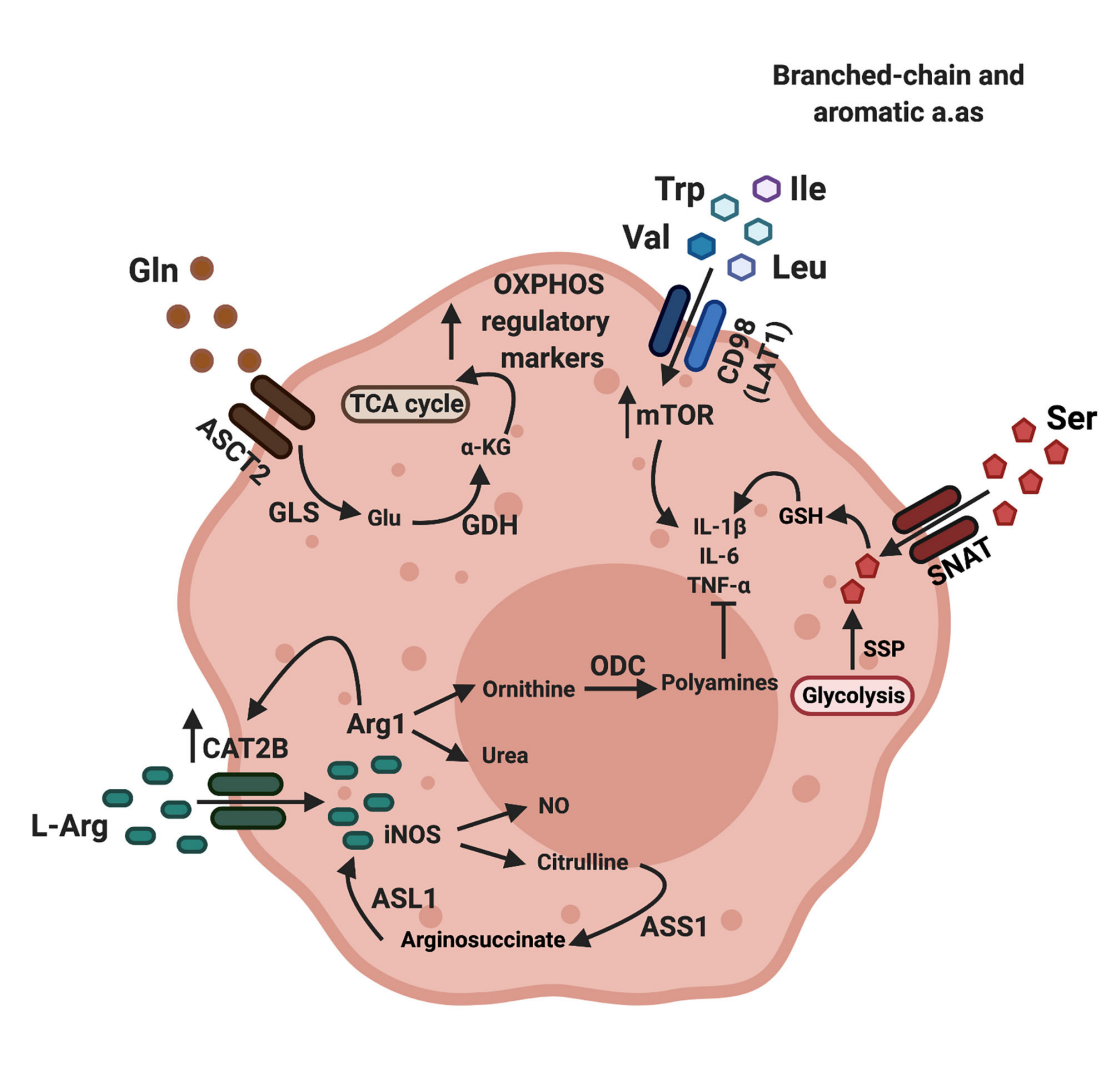
Amino acid transporters regulate amino acid availability and macrophage polarization. Arginine enters macrophages through cationic amino acid transporters (CAT), while glutamine and aromatic or branched chain amino acids enter macrophages though the neutral amino acid transporters ASCT2 (alanine serine cysteine transporter 2) and LAT1 (large amino acid transporter 1) respectively. Arginine can be catabolized by either arginase-1 (Arg1) or nitric oxide synthase (iNOS). Citrulline, a metabolite produced by the action of iNOS can be converted back to arginine by the enzymes arginosuccinate synthetase (ASS1), which converts citrulline to arginosuccinate and arginosuccinate lyase (ASL1), which converts arginosuccinate back to arginine. The net positive flow and accumulation of amino acids in macrophages through amino acid transporters result in increased mTOR activity and production of pro-inflammatory cytokines such as IL-1b and TNFa. The product of arginase 1-mediated arginine catabolism, ornithine, is converted to polyamines through the action of ornithine decarboxylase (ODC). When Glutamine (Gln) enters macrophages, it is converted to glutamate (Glu) by glutaminase (GLS), and in turn, glutamate will be converted to a-ketogluterate (a-KG) by glutamate dehydrogenase (GDH). Ornithine, polyamines and a-ketoglutarate directly inhibit synthesis of inflammatory macrophage markers and promote the production of regulatory macrophage markers by feeding into the TCA cycle, leading to a regulatory phenotype (7, 21, 22). Image created using biorender.

Sustained Arg1 and Nos2 activity require a steady transport of arginine into the cell. The cationic amino acid transporter family, which includes CAT1, CAT2A, CAT2B and CAT3, is the major transporter of arginine into cells (**Figure 1**). In particular, CAT2B is thought to be the most efficient in the transport of arginine into cells. It was reported that in macrophages, Arg1, but not Nos2 induces expression of *Cat2b (* 23 *)*. In MDSCs however, *Cat2b* expression is induced by both Arg1 and Nos2 suggesting a dichotomy in the biology (23). Moreover, *Cat2^-/-^* M-MDSCs or PMN-MDSCs derived from ascites of prostate tumor-bearing mice displayed a significantly reduced ability to inhibit T cell proliferation in *in vitro* assays (24). Similarly, using a thymoma tumor model, it was shown that anti-tumor activity of adoptively transferred tumor antigen specific CD8^+^ T cells was significantly enhanced in *Cat2^-/-^* vs. wild-type mice. This was the result of decreased ability of *Cat2^-/-^* MDSCs to suppress T cell function and proliferation *in vivo* (24).

Arginine catabolism produces the polyamines putrescine, spermidine and spermine. Downstream of Arg1, ornithine decarboxylase (Odc) catalyzes the rate-limiting step that converts ornithine into putrescine which is further metabolized into spermidine and spermine (25). This key pathway of arginine metabolism may provide support for tumor growth. For example, increased polyamine levels and activation of Odc were correlated with tumorigenesis and increased tumor growth in melanoma, mesothelioma, colon and prostate cancer (26, 27). In macrophages, polyamines suppress the production of pro-inflammatory cytokines such as IL-1β, IL-6 and TNF-α, resulting in a more tolerogenic phenotype (28, 29). Moreover, myeloid specific deletion of Odc resulted in more severe *H. pylori* induced gastritis or *C. rodentium* induced colitis while bacterial burden remained unchanged, suggesting enhanced responsiveness to inflammatory stimuli (30).

## Tryptophan Consumption in Immunity

**Figure 2:**
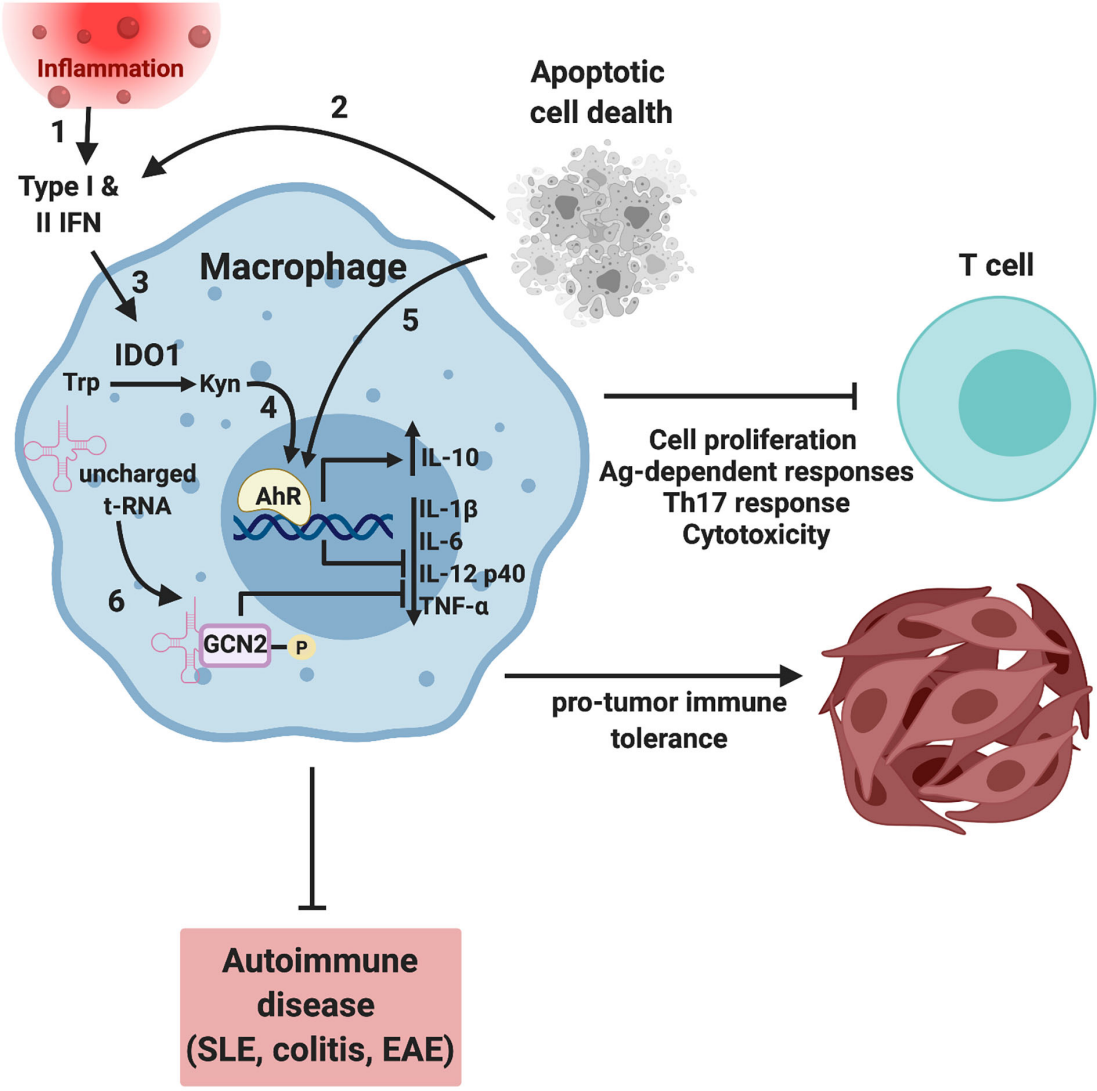
Tryptophan metabolism regulates macrophage function by AhR and GCN2-dependent mechanism. Inflammation (1) and/or apoptotic cell phagocytosis (2) induces interferon expression that drives expression of the interferon responsive gene (ISG) IDO1 (3). IDO-dependent enzymatic consumption of tryptophan results in the accumulation of tryptophan catabolites such as L-kynerunine that bind to and activate the transcription factor AhR (4), driving a tolerogenic macrophage phenotype. In addition, apoptotic cells can activate AhR in an IDO-independent, TLR9-dependent mechanism (5) (3). In parallel, amino acid starvation results in increased uncharged tRNAs and translational stress driving activation of GCN2 (6). GCN2 activity drives a more regulatory, less inflammatory macrophage phenotype suppressing T cell activation, proliferation, and Th17 responses. In the context of autoimmune disease such as lupus, colitis and autoimmune encephalomyelitis (EAE), decreased inflammation results in a more favorable disease outcome with less severe symptoms and increased survival (4, 33–36). Conversely, amino acid depletion results in a tolerogenic tumor immune microenvironment, tumor growth and decreased survival (37). Image created using biorender.

The role of oxidative tryptophan catabolism in immune regulation was first identified in maternal-fetal tolerance over 20 years ago (31). In mammals this process is mediated by two closely related indoleamine-pyrrole 2,3 dioxygenase enzymes [indoleamine 2,3 dioxygenase (IDO)1 and IDO2] and the unrelated enzyme tryptophan 2,3 dioxygenase (TDO) (32). The *IDO1* gene is primarily expressed by myeloid cells and stroma in response to inflammatory immune signals (although it can be expressed by tumor cells), (**Figure 2**) whereas *IDO2* and *TDO* are largely unresponsive to immune stimuli and have a broader expression pattern (38); thus, this discussion will focus on IDO1. IDO1 catalyzes the first and rate-limiting step of oxidative tryptophan consumption to produce N-formyl-L-kynerunine, which can be catabolized further to immunologically reactive intermediates such as kynerunic acid and 3-hydroxyanthralinic acid (39). Our work has shown that IDO1 expression is induced in macrophages in response to apoptotic cell phagocytosis (4). Importantly apoptotic cell-induced IL-10 production was dependent on IDO1 activity and when *Ido1* was deleted or inhibited pharmacologically apoptotic cell-exposure induced a significant proinflammatory cytokine response with increased TNFα, IL-6, and IL-12 production (4). This increased inflammatory activity caused a breakdown in immunologic tolerance to apoptotic cell-associated material with augmented serum and cellular autoreactivity and exacerbation of disease pathology in animal models of systemic lupus erythematosus (4). This data clearly showed that IDO1 is an important driver of tolerance towards autoantigens with implications for autoimmune disease and cancer.

Research examining the molecular underpinnings of IDO1-mediated immune regulation has focused on the effect of amino acid consumption (i.e. amino acid starvation stress) and the production of effector catabolites (see **Figure 2**). IDO1-generated N-formyl-L-kynurenine is further catabolized by aryl formamidase to form L-kynurenine (L-Kyn) (40). L-Kyn is a key product of IDO1 catabolism of tryptophan and, together with other downstream catabolic products (e.g. cinnabaric acid), is a regulator of immunity *via* binding to the aryl hydrocarbon receptor (AhR) (33). AhR is a cytoplasmic receptor/transcription factor that serves a key role in immune function, particularly at mucosal sites (33). While AhR activity can promote inflammation, the evidence suggests it is primarily a driver of immune-regulatory responses (33). In T cells, exposure to L-Kyn promotes FOXP3^+^CD4^+^ T reg development from naïve precursors and may potentiate their function (25) and increases PD-1 expression on effector T cells limiting function (41). Similarly, AhR can potently influence macrophage phenotype. For example, AhR null mice exhibit increased susceptibility to LPS-induced toxicity due to elevated STAT1 signaling and IL-6 production in macrophages (42). Likewise, we have demonstrated that AhR is a requisite driver of IL-10 expression in mouse and human macrophages, while macrophage-specific deletion in mice causes autoimmune disease late in life (3). In the same vein, mice with a deletion of AhR in microglia develop more severe autoimmune encephalomyelitis (EAE) symptoms with increased expression of pro-inflammatory genes and a loss of IL-10 and TGFα expression (34). The linkage to tryptophan consumption is clear as a tryptophan-deficient diet leads to worse EAE symptoms, while restoring dietary tryptophan reverses this effect in MICROGLIAL-AhR sufficient but not deficient mice, suggesting tryptophan metabolites exert their function by activating AhR in the microglia (34). These studies underscore the importance of tryptophan metabolism and its effectors such as AhR in controlling the inflammatory functions of macrophages.

AhR function in MDSCs has not been examined in detail. However, MICROGLIAL-AhR potently impacts hematopoietic progenitor development driving expansion of precursors (43) and in acute myeloid leukemia AhR signaling may promote differentiation of leukemic stem cells (44). Since at least some MDSC populations expand as a result of emergency granulopoiesis (45), AhR signaling may impact MDSC expansion and differentiation by causing proliferation and differentiation of hematopoietic precursors. Dioxin (i.e. 2,3,7,8-tetrachlorodibenzo-*p*-dioxin) is an environmental pollutant that is a high affinity AhR ligand and was used to originally identify the *AHR* gene (33). Treatment of mice with dioxin increases MDSC expansion and mobilization in an AhR-dependent mechanism further indicating of a role for AhR in MDSC expansion (46). However, it remains to be seen if physiologic AhR ligands like L-Kyn exhibit a similar effect.

General control nonderepressible 2 (GCN2) is an ancient protein found in all eukaryotes that regulates cellular function to match nutrient supply, however in immune cells it serves an additional purpose controlling inflammatory function (38). GCN2 is activated by ribosome translational stress resulting from amino acid deficiency (38) and functions by phosphorylating the α subunit of eucaryotic initiation factor (eIF)2, slowing ribosome assembly and cap-dependent translation (47). Moreover, as a result of altered codon usage when eIF2α is phosphorylated, GCN2 also drastically increases translation of ATF4, a key transcription factor required for GCN2-dependent responses (48). GCN2 can be activated by IDO1-driven tryptophan consumption and is an important component of the biologic effects of IDO1 induction in immune cells. For example, IDO1 fails to induce IL-10 protein in *Gcn2* knock out macrophages due to alterations in ribosome association with cytokine mRNA transcripts (35). Moreover, macrophage GCN2 activation inhibits IL-1β and reactive oxygen species production, at least partially by limiting inflammasome complex assembly, worsening colitis in mice (36). Notably, restricting protein in the diet protects *WT* but not *Gcn2^-/-^* mice against colitis, suggesting amino acid starvation signaling pathways, by activating GCN2, dampen gut inflammation (36). In the same vein, we recently reported that GCN2 is a mechanistic driver of macrophage function in the tumor microenvironment (37). The effect was dependent on ATF4, however DNA binding analysis suggested that in tumor macrophages ATF4 was primarily impacting expression of genes involved in metabolism (37). When we examined the transcriptomes and metabolic profile of tumor macrophages lacking GCN2 there was no change in expression of genes involved in glycolysis, however, there was a significant reduction in expression of oxidative metabolism genes. At the functional level, this resulted in a reduction of oxidative respiration in tumor macrophages that shifted polarization to a more proinflammatory glycolytic profile (37).

In MDSCs much less is known regarding the role of GCN2 and function. However, we found that GCN2 activity is requisite for suppression of T cell maturation (37). This is likely attributable to significant reduction in expression of several key suppressive mediators in *Gcn2^-/-^* MDSCs compared to wildtype MDSCs (37). Metabolically, the impact of GCN2 deletion on MDSCs is pronounced, drastically reducing basal mitochondrial respiration and spare respiratory capacity suggesting the lack of GCN2 severely compromises MDSCs oxidative metabolic function. Thus, data from our lab and others have clearly shown that GCN2 is a key driver of macrophage and MDSC immune-regulatory function, impacting phenotype *via* direct regulation of cytokine mRNA translation, and indirectly by alteration of metabolic function. It is important to note that GCN2 can be activated by paucity of any amino acid and is not a specific function of tryptophan depletion per se. Thus, GCN2 effects on myeloid immunity likely extend far beyond the biology of IDO1.

## Glutamine

Glutamine is the most abundant free amino acid in the body, providing intermediates for many metabolic pathways and a key source of substrates for diverse physiological functions including production of purines and pyrimidines, conversion of ammonia into urea, acid-base balance, and TCA cycle intermediates (49). Inflammatory macrophages have a break in the TCA cycle that increases succinate in the cell promoting IL-1β production. Recently it was reported that Nos2 inhibits aconitase 2 and pyruvate dehydrogenase limiting entry of metabolites in the TCA cycle and driving glutamine to α-ketoglutarate conversion *via* anaplerosis (50), a key source of succinate production in inflammatory macrophages (9, 51). This suggests the mode of arginase consumption is linked to glutamine metabolism in macrophages.

One of the metabolic hallmarks of alternatively activated macrophages is their use of oxidative phosphorylation for energy production (8). This metabolic shift is driven by IL-4 and requires glutaminolysis-driven production of α-ketoglutarate and alteration in the α-ketoglutarate/succinate ratio in macrophages promoting an immune-suppressive phenotype (52). Glutamine is not required for inflammatory polarization, however glutamine deprivation decreased levels of regulatory macrophage markers CD206, CD301, and Relmα suggesting a direct role in regulatory polarization (53). In regulatory macrophages, peroxisome proliferator-activated receptor γ (PPARγ) was found to be necessary for usage of glutamine in the TCA cycle and PPARγ-deficient macrophages exhibit defective glutamine with decreased OXPHOS and a more inflammatory phenotype. Glutamine metabolism was also found to be important for the recruitment and function of MDSCs and TAMs at tumour sites. Blocking glutamine metabolism resulted in decreased tumour size and MDSC infiltration in mammary tumors resulting from increased caspase-3 mediated apoptosis in MDSCs. In addition, TAMs exhibited a more pro-inflammatory tumoricidal phenotype, with increased TNFα secretion, activation of Nf-κB and a reduction in STAT3 signaling and IL-10 production (54).

## Branched Chain Amino Acids

In addition to glutamine the branched chain amino acids (i.e. BCAA-leucine, isoleucine, valine) are key drivers of metabolic programming and function in myeloid cells. BCAAs are a major carbon source for metabolism, glutamine production, and serve as substrates for generation of acetyl-CoA and succinyl-CoA for the TCA cycle (55). CD98 transports BCAA and aromatic amino acids into the cell and consists of 2 subunits, Slc7a5 or CD98 light chain (CD98lc) and Slc3a2 or CD98 heavy chain (CD98hc). In human monocytes and macrophages, Slc7a5-mediated leucine influx drives mTORC1 activation, with increased IL-1β and TNFα production (56). Moreover, increased Slc7a5 expression in circulating monocytes was linked to rheumatoid arthritis and correlated with severity of the disease (56). In a dextran-sulfate induced model of colitis, selective deletion of *Slc3a2* in CX3CR1+ monocytes and macrophages of the colonic lamina propria led to increased apoptosis and reduced expression of MHCII, reducing the severity of the disease (57). Moreover, deletion of branched-chain amino transferase (BCAT)1 reduced inflammatory macrophage polarization (58). BCAT1 transaminates BCAA to begin catabolism to coenzyme A derivatives and glutamate (32, 59, 60) providing key intermediates for the TCA cycle. Since mTOR signals and TCA cycle intermediates (e.g. NAD+, citrate, succinate) are potent drivers of inflammatory macrophage function (61), this suggests sustained import and consumption of BCAAs provides important signals in activated macrophages promoting and supporting inflammatory function.

## Serine

Serine is a non-essential amino acid that can be obtained from exogenous sources through amino acid transporters ASCT2 or SNAT or synthesized *de novo* through the serine synthesis pathway (SSP) (62). The SSP is a branch of glycolysis that allows serine to be synthesized from the glycolytic or gluconeogenic intermediate 3-phosphoglycerate through three catalytic reactions, the first of which being catalysed by phosphoglycerate dehydrogenase (PHGDH). Serine can modulate the SSP through its interaction with pyruvate kinase isoform M2 (PKM2), which catalyses the last step in glycolysis. When serine is at normal physiological levels in cells, it binds to and activates pyruvate kinase isoform M2 (PKM2), leading to production of pyruvate from 3-phosphoglycerate. In contrast, when serine levels are low, PKM2 activity decreases, allowing for 3-phosphoglycerate to be used for serine production. Serine is an important contributor to one-carbon metabolism and is essential for nucleotide synthesis and redox balance through the replenishment of NADPH *via* the folate cycle as well as to produce s-adenosylmethionine (SAM), a co-substrate required for methylation reactions through the methionine cycle (63).

Recent studies revealed that serine metabolism is a key contributor to IL-1β production in inflammatory macrophages (64, 65). Serine synthesis is increased in macrophages after LPS stimulation serving as a carbon source for glycine and subsequent glutathione (GSH) production. Importantly macrophages cultured in serine-free conditions exhibited a marked reduction in GSH following LPS exposure suggesting that serine is a critical substrate for early GSH production (64). The role of serine in early GSH synthesis post-LPS stimulation is distinct from Nrf2-driven redox responses manifesting at later time points after LPS exposure. This suggests altered serine production/metabolism provides a rapid, transcriptionally independent, mechanism to boost GSH production immediately after LPS exposure for redox balance thereby promoting IL-1β expression. Moreover, both glucose and serine-derived one-carbon metabolism synergize to enhance SAM production in LPS-stimulated macrophages driving increased trimethylation at H3K36, a signature that is linked to transcriptional activation and IL-1β mRNA production (65). Thus, the data suggests the serine production and metabolism are a key component of macrophage inflammatory responses. Much less is known regarding serine metabolism in MDSCs. However, a recent report demonstrated that inhibiting glutamine metabolism with a small molecule antagonist drives MDSC inflammatory maturation that is associated with significant increases in intratumoral glycine and serine metabolm (54). While the report did not assess MDSCs specifically in this context, it is tempting to speculate that in the absence of glutamine metabolism, increased serine metabolism may contribute to inflammatory maturation. However this remains to be tested.

## Closing Remarks

Amino acid metabolization is fundamental to myeloid polarization, altering and reinforcing the metabolic wiring that drives functional phenotypes. However, it is only recently that we have begun to understand the mechanistic underpinnings of these processes. With the emerging granular knowledge of amino acid usage and its role in immunity comes the opportunity to target these pathways in therapeutic settings. For example, blocking glutamine metabolism reduces IDO activity in tumor cells, promotes MDSC apoptosis, and shifts macrophages to a proinflammatory phenotype driving anti-tumor immunity (54). Similarly, blocking amino acid consuming enzymes like Arg1 may have the combined benefit of directly abrogating the immune regulatory effect of the enzyme while rewiring the immune cells towards a more desired phenotype for lasting impact (66, 67). However, care must be taken with this approach. There was significant early enthusiasm targeting IDO for cancer immunotherapy, driven by a broad set of experimental and early clinical data that was supportive of the target and inhibitors. However, the recent, widely publicized failure of the ECHO-301 trial, a combination of the IDO-inhibitor Epacadostat and pembrolizumab (αPD-1) in advanced melanoma patients, illustrates the inherent challenges in targeting these pathways (68). An approach that has the potential to bypass innate problems with drug bioavailability, toxicity, and off target effects is dietary intervention and restriction. Indeed there is a long history in the literature showing that restriction of protein or amino acids has therapeutic effect in a wide range of inflammatory conditions (69–71). However, clinical trials to test the efficacy of low protein diet on outcomes in human disease have been less clear-cut, likely due to the inability of patients to adhere to such restrictive dietary regiments (72, 73). Thus it is clear that while our knowledge of amino acid metabolism and myeloid function has greatly expanded, there is still much to learn before we can utilize this knowledge for therapeutic benefit.

## Author Contributions

MH and TM conceived and wrote the manuscript. All contents are agreed upon by all authors. All authors contributed to the article and approved the submitted version.

## Funding

The work described in this review supported by NIH grants R01AR067763, R01CA190449, the Terry Fox Research Institute New Frontiers Program, and the Canadian Institutes of Health Research Grants 162114 and 436605 (TM).

## Conflict of Interest

The authors declare that the research was conducted in the absence of any commercial or financial relationships that could be construed as a potential conflict of interest.

## Publisher’s Note

All claims expressed in this article are solely those of the authors and do not necessarily represent those of their affiliated organizations, or those of the publisher, the editors and the reviewers. Any product that may be evaluated in this article, or claim that may be made by its manufacturer, is not guaranteed or endorsed by the publisher.
